# Asymptomatic Symmetric Bilateral Mandibular Tori: A Case Study

**DOI:** 10.7759/cureus.66411

**Published:** 2024-08-07

**Authors:** Unnati Shirbhate, Pavan Bajaj, Manoj Patil

**Affiliations:** 1 Department of Periodontics, Sharad Pawar Dental College and Hospital, Datta Meghe Institute of Higher Education and Research, Wardha, IND; 2 Department of Public Health, Sharad Pawar Dental College and Hospital, Datta Meghe Institute of Higher Education and Research, Wardha, IND

**Keywords:** benign, oral lesions, torus palatinus, bony exostosis, bony outgrowth, mandibular tori

## Abstract

Tori are reactive or developmental localized overgrowths of alveolar bone that are not cancerous. A thin, weakly vascularized mucosa surrounds a densely cortical, low-density mass of bone marrow known as tori or exostosis. Tori are more frequently observed in middle age. Both the maxilla (torus palatinus) and the mandible (torus mandibularis) exhibit tori. Difficulty in speaking and other issues are common obstacles associated with tori. Tori range in diameter from a few millimeters to several centimeters. Surgical excision of tori is the mainstay of treatment for large tori obstructing speech, mastication, or tongue position. The following case study includes a 36-year-old male patient with an association of mandibular canine and premolar regions with bony outgrowth.

## Introduction

Torus mandibularis (TM) is a benign, hamartomatous bony, non-tender extension located on the lingual side of the jaw, primarily in the canine or premolar region, above the point of mylohyoid muscle attachment. Bilateral tori tend to be present more frequently. Typically, tori are asymptomatic and found by accident. The prevalence differs significantly among ethnic groups: Asian and Inuit populations have a higher frequency, whereas White and Black populations have lower prevalence (about 8% and 16%, respectively). Males are more likely to have TM than females [1]. Stomatognathic system morphological and functional changes were facilitated by mandibular tori [2].

In three places in the oral cavity, the torus palatinus (TP), TM, and torus maxillaris are common, paucisymptomatic bony outgrowths. Their histological features are not specific, as they are typically characterized by trabecular bone, which is present mainly in normal cortical bone [3]. Instead of developing a simian shelf, humans have strengthened the weakest portion of their jaw by creating an external chin. As a result, as the mandibular body buckles medially due to a combination of muscle compression and tooth orientation controlled by the maxilla, the morphology of the mandible localizes torus development to the premolar area [4].

Because parafunctional activity concentrates mechanical stresses in the area where TMs usually occur, it may be the cause of TM development. Consequently, TM will be more common in mandibular geometries that promote stress concentration, such as square-shaped mandibles. The main causes of TM are believed to be environmental, including bruxism, vitamins involved in bone health, such as vitamins A and D, and supplements high in calcium. However, genetics certainly plays a significant role [5,6].

## Case presentation

**Figure 1:**
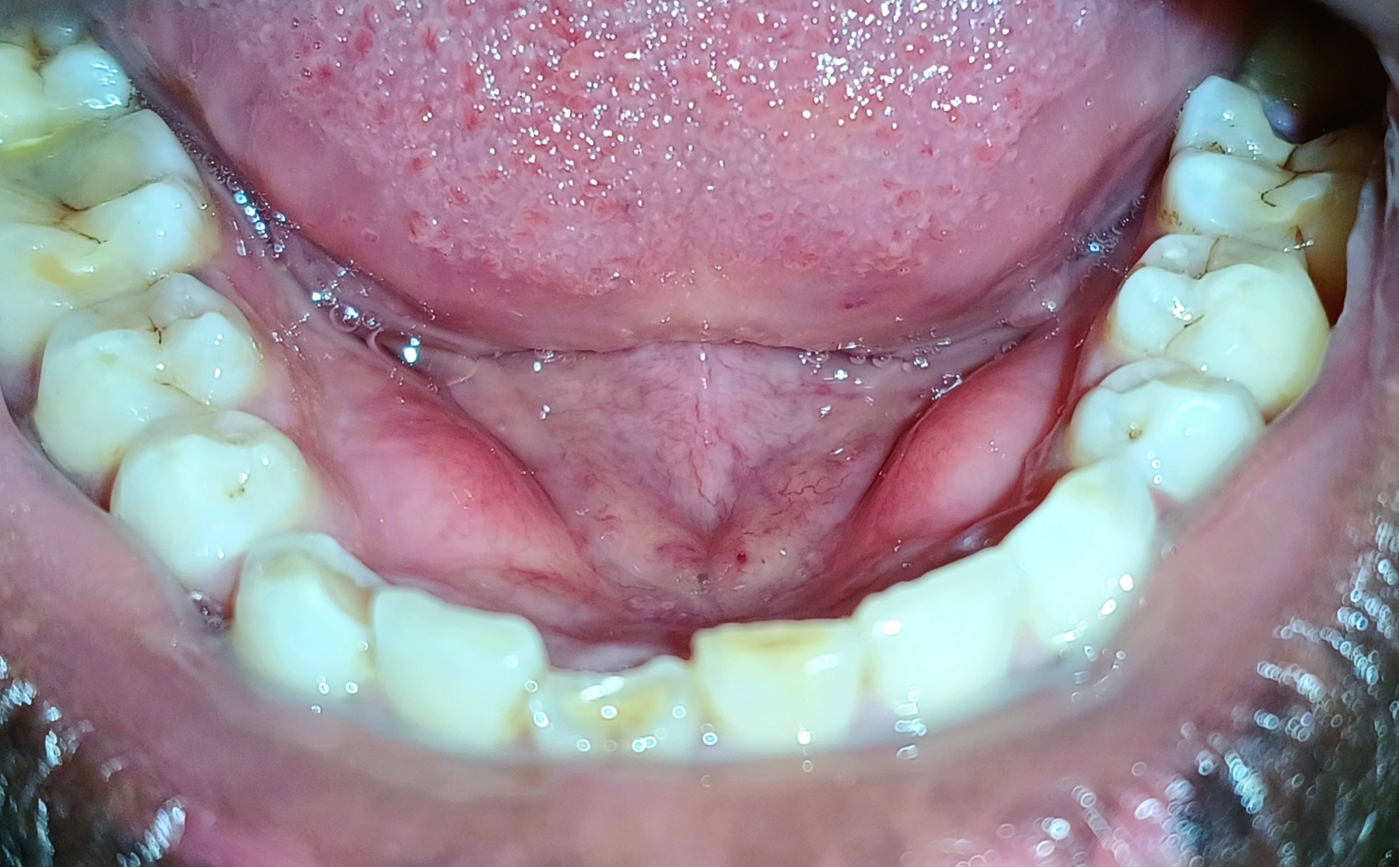
Bilateral mandibular tori present with canine and premolar regions

The following 36-year-old male patient reported to the Sharad Pawar Dental College and Hospital, Department of Periodontics, complaining of slowly progressing hard swelling in the mandibular lingual region. Bilateral bony outgrowths, shown in Figure 1, associated with the canine, first, and second premolar regions, were noted. Clinical examination revealed bilateral mandibular tori related to the floor of the mouth; these were non-tender and symmetrical. Intraoral hard tissue examination revealed generalized attrition, which might be a suspected factor causing bruxism, leading to attrition and mandibular tori in this case. The bony swellings were asymptomatic, but as they grew, the patient had difficulty stabilizing the placement of his tongue. Since the tori were painless and the symptoms did not cause any deformity or functional problems, surgical removal was avoided.

## Discussion

Although the exact cause of tori is unknown, many authors believe a significant hereditary component exists. There are significant differences in the reported prevalence of mandibular tori among ethnic groups. More than 90% of cases involve bilateral mandibular tori. Except for certain edentulous patients, in whom tori may impair the fit of dental prostheses, tori often do not cause any symptoms despite their potential for sluggish growth. The majority of tori don't need therapy. Large tori may be cut or removed, particularly if they interfere with receiving prosthetic care [6]. In addition to dietary, gender, and ethnic characteristics, Valentin et al. emphasize the significance of mechanical overload in the recurrence of exostoses, which may cause recurrence in the patient [7]. The tori can be of two types: TP and TM and can present in various shapes such as regular or irregular, flat, spindle-shaped, or nodular. Mandibular tori are classified as unilateral or bilateral solitary, unilateral or bilateral multiple, and bilateral combined [2,5]. The patient was speculated to have bruxism, which leads to attrition and clenching habits, contributing to tori development in this case study - a factor rarely considered in tori cases. To understand the exact cause and its symptoms, knowledge about the patient's habits should be carefully assessed by clinicians. The tori were asymptomatic in this case until now; however, the patient has been educated about their development and his habits, so he can improve his oral hygiene practices and be aware of any potential secondary effects in the future.

One of the biggest challenges is identifying the parameters linked to the probability of recurrence. Bertazzo-Silveira et al.'s analysis shows that tori, particularly TM, may be linked to inconsistent tooth wear [8]. There is insufficient data to support or refute the link between tori and further bruxism signs and symptoms. The condition known as TM, or mandibular exostoses, affects many patients. Most of these benign, asymptomatic bone outgrowths remain unaltered throughout the patient's life. Nonetheless, it is often necessary to remove the tori [9-11]. TM grows slowly and cannot change malignantly. Until a TM becomes large enough to impede the function of mastication, therapy is typically not necessary; in that case, surgery is required [12-14].

## Conclusions

The following case study demonstrates that the mandibular tori are slowly progressing, and many are often asymptomatic. However, the cause needs to be addressed to avoid damaging the phonetics and functioning of the stomatognathic system as early as possible. Patient awareness of the etiology of tori should be emphasized in many cases to address functional demands.
